# Death-associated protein 3 is overexpressed in human thyroid oncocytic tumours

**DOI:** 10.1038/sj.bjc.6605111

**Published:** 2009-06-16

**Authors:** C Jacques, J-F Fontaine, B Franc, D Mirebeau-Prunier, S Triau, F Savagner, Y Malthiery

**Affiliations:** 1 INSERM, U694, Centre Hospitalier Universitaire d'Angers, Laboratoire de Biochimie et Biologie Moléculaire, F-49033 Angers, France; 2 Faculté de Médecine, Université d'Angers, rue haute de Reculée, F-49045 Angers, France; 3 Max-Delbrück-Center for Molecular Medicine, D-13125 Berlin, Germany; 4 Service d'Anatomie Pathologique, Hôpital Ambroise Paré (APHP), F-92104 Boulogne, France; 5 Faculté de Médecine Paris Ile de France Ouest, Université de Versailles Saint Quentin en Yvelines, 9 bd d'Alembert, bât. François-Rabelais, F-78280 Guyancourt, France; 6 Laboratoire de Biochimie, CHU, 4 rue Larrey, F-49033 Angers, France; 7 Service d′anatomie pathologique, CHU, 4 rue Larrey, F-49033 Angers, France

**Keywords:** thyroid, oncocytic tumours, mitochondrion, DAP3, transcriptional regulation

## Abstract

**Background:**

The human death-associated protein 3 (hDAP3) is a GTP-binding constituent of the small subunit of the mitochondrial ribosome with a pro-apoptotic function.

**Methods:**

A search through publicly available microarray data sets showed 337 genes potentially coregulated with the *DAP3* gene. The promoter sequences of these 337 genes and 70 out of 85 mitochondrial ribosome genes were analysed *in silico* with the *DAP3* gene promoter sequence. The mitochondrial role of DAP3 was also investigated in the thyroid tumours presenting various mitochondrial contents.

**Results:**

The study revealed nine transcription factors presenting enriched motifs for these gene promoters, five of which are implicated in cellular growth (ELK1, ELK4, RUNX1, HOX11-CTF1, TAL1-ternary complex factor 3) and four in mitochondrial biogenesis (nuclear respiratory factor-1 (NRF-1), GABPA, PPARG-RXRA and estrogen-related receptor alpha (ESRRA)). An independent microarray data set showed the overexpression of *ELK1*, *RUNX1* and *ESRRA* in the thyroid oncocytic tumours. Exploring the thyroid tumours, we found that DAP3 mRNA and protein expression is upregulated in tumours presenting a mitochondrial biogenesis compared with the normal tissue. *ELK1* and *ESRRA* were also showed upregulated with *DAP3*.

**Conclusion:**

*ELK1* and *ESRRA* may be considered as potential regulators of the *DAP3* gene expression. DAP3 may participate in mitochondrial maintenance and play a role in the balance between mitochondrial homoeostasis and tumourigenesis.

Mitochondria play a major role in life and death of cells, ensuring most of the cellular ATP synthesis through oxidative phosphorylations (OXPHOS) and participating in the induction of apoptosis ((Regula *et al*, 2003) for review). Mitochondrial defects have been implicated in a wide variety of pathologies, including degenerative diseases and cancer, as well as in physiological processes such as aging (Schapira, 2006).

Mitochondria possess their own genome; mitochondrial DNA (mtDNA) encodes 37 genes corresponding to two rRNAs, 22 tRNAs and 13 mRNAs. The 13 mRNAs, translated on mitochondrial ribosomes, encode for essential catalytic components of four of the five OXPHOS complexes. The mitochondrial ribosome is a ribonucleoproteic complex composed of two asymmetric subunits, the large subunit containing a 16S rRNA molecule associated with 52 proteins, and the small subunit containing a 12S rRNA molecule associated with 33 proteins. The 85 mitochondrial ribosomal proteins (MRP) are encoded by nuclear genes, translated in the cytoplasm and addressed by mitochondrial signals to the appropriate mitochondrial compartment, where they associate with the two rRNA molecules ((O'Brien, 2002; Mears *et al*, 2006) for review).

The death-associated protein 3 (DAP3) is one of the constituents of the small subunit of the mitochondrial ribosome that has no counterpart in bacterial or cytoplasmic ribosomes (Cavdar Koc *et al*, 2001a, 2001b; Suzuki *et al*, 2001a, 2001b; O'Brien *et al*, 2005). Multiple roles have been assigned to DAP3; first identified as a pro-apoptotic factor, this protein is suspected of interacting with the TNF-related apoptosis-inducing ligand (TRAIL) receptors and the Fas-associated death domain protein (FADD) in the cytosol (Kissil *et al*, 1995, 1999; Miyazaki and Reed, 2001).

Knocking down *DAP3* by small interfering RNA (siRNA) or short hairpin RNA (shRNA) reduces fragmentation of the mitochondrial network, increases resistance to oxidative stress and decreases the production of intracellular reactive oxygen species (ROS) (Mukamel and Kimchi, 2004; Murata *et al*, 2006). The treatment of HeLa cells with a *DAP3* siRNA led to a five-fold reduction of mitochondrial respiration. Homozygous *DAP3* –/– mouse embryos, which died *in utero* at 9.5 days of gestation on an average, had low levels of mitochondrial proteins encoded by the mtDNA (Kim *et al*, 2007). These results indicate that DAP3 is essential to mammalian cells because of its contribution to mitochondrial maintenance; however, the function of DAP3 in mitochondrial protein synthesis remains to be elucidated.

The implication of DAP3 in apoptosis suggests a modulation of the expression of the protein in pathological contexts such as the development of tumours. First explored in glioblastoma multiform (GBM) cells, the *DAP3* mRNA and protein were found to be overexpressed in the invasive GBM cells (Mariani *et al*, 2001). In thymoma, the *DAP3* mRNA level was positively correlated with the stage of the disease defined in the World Health Organisation (WHO) classification (Sasaki *et al*, 2004). In contrast, a study on senescence induced by oxidative stress in cells showed reduced DAP3 expression (Murata *et al*, 2006).

In this study, we explored the function of DAP3 in mitochondrial translation analysing multi-source microarrays and genomic data to identify potential coregulated genes and regulators of the *DAP3* gene. We choose to investigate the thyroid tumours on the basis of their various mitochondrial content, and, in particular, the thyroid oncocytoma, a mitochondrial-rich thyroid tumour characterised by an oxidative metabolism (Savagner *et al*, 2003; Baris *et al*, 2004). In oncocytoma, the large majority of the cells show extensive mitochondrial biogenesis; these tumours thereby constitute an interesting model for the study of the expression and localisation of DAP3, as well as its function in the mitochondrial ribosome. We studied the expression of the DAP3 mRNA and protein in thyroid tumours using quantitative real-time polymerase chain reaction (qRT–PCR) and immunohistochemistry (IHC) techniques. Our results suggest that the *DAP3* gene is modulated by major transcriptional regulators of mitochondrial biogenesis, and coregulated with genes of the small subunit of the mitochondrial ribosome.

## Materials and methods

### Materials

All the tissue samples studied belong either to the tumours collection of the Laboratoire d'anatomie pathologique, Centre Hospitalier Universitaire d'Angers, France or of the Laboratoire d'Anatomie Pathologique, Hôpital Ambroise Paré (APHP), Boulogne, France. For the first analysis by quantitative PCR (qPCR), 40 thyroid samples were used representing normal thyroid (NT, 10 samples) and three types of thyroid tumours (10 follicular thyroid adenomas (FTA), 10 papillary thyroid carcinoma (PTC) and 10 oncocytic thyroid tumours (OTT)). A second analysis was carried out on samples from 17 other patients with oncocytic thyroid adenomas (OTA, 12) or oncocytic thyroid carcinomas (OTC, 5). The tumours were diagnosed according to the WHO classification (DeLellis *et al*, 2004). The normal paired thyroid samples, taken at a distance from the tumours, were examined in the histology laboratory before being adopted for controls. All the samples rendered anonymous, that is, with patient identifiers deleted before the study, were deep-frozen in liquid nitrogen immediately after surgery and conserved at −80 °C.

The IHC studies carried out on formalin-fixed, paraffin-embedded tissue sections concerns 100 thyroid tumour samples and 61 normal counterparts. The cases enroled in the study were distinct from the groups participating in the qPCR analyses. The tumours were classified as nine OTC, thirty-four PTC, four poorly differentiated carcinomas, five follicular thyroid carcinomas (FTC), three non-medullary thyroid tumours of uncertain malignancy potential (TUMP), thirty-seven OTA and eight FTA.

### Bioinformatic analysis

**Figure 1:**
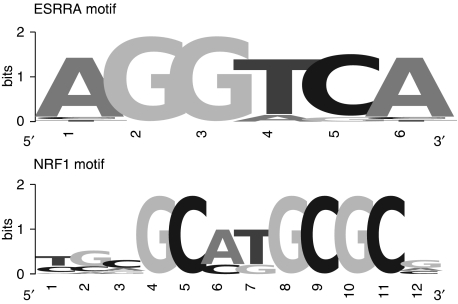
Estrogen-related receptor alpha (ESRRA) and nuclear respiratory factor-1 (NRF-1) position–weight matrices. The graphical representation of the ESRRA and NRF-1 motifs visualised using the WEBLOGO web server (http://weblogo.berkeley.edu/) shows a six-position–weight matrix for ESRRA and a 12-position–weight matrix for NRF-1 (x-axis). For each position, nucleotides are represented according to their relative frequency, the overall height indicating the conservation of the sequence (y-axis).

We queried the TMM web server (www.bioinformatics.ubc.ca/tmm) for significantly correlated *DAP3* coexpressed genes, that is, the genes that were correlated in at least three out of 100 microarray data sets (Lee *et al*, 2004). We also used the 85 mitoribosomal genes for sequence analysis. Gene promoter sequences were extracted by the Promoser web server from −1000 to +1000 nucleotides starting from the transcription start site (TSS) (Halees *et al*, 2003). Promoser retrieved 69 out of 331 sequences of the DAP3 coexpressed genes, and 71 out of 85 sequences of the mitoribosomal genes. We collected 123 transcription factor binding sites (TFBS) motifs from the Jaspar database (Sandelin *et al*, 2004). We added two position–weight matrices to this collection: for the transcriptional factor nuclear respiratory factor-1 (NRF-1), we aligned nine sequences of known NRF-1 binding sites (Au and Scheffler, 1998; Elbehti-Green *et al*, 1998; de Sury *et al*, 1998; Hirawake *et al*, 1999); for the estrogen-related receptor alpha (ESRRA), the position–weight matrix was described by Sladek *et al* (1997) (Figure 1).

The TFBS overrepresentation in promoter sequences was investigated with the Clover program (http://zlab.bu.edu/clover/) using the 125 TFBS motifs and two background models (Frith *et al*, 2004). The first consisted of 6461 randomly chosen gene promoter sequences of the human genome from −1000 to +1000 nucleotides starting from the TSS, and the second was composed of 27 555 sequences from CpG island regions. The selected threshold of significance was *P* ⩽0.05 simultaneously with both background sets.

The *DAP3* gene promoter (−1000 to +1000 nucleotides starting from the TSS) was also analysed using the collection of 125 TFBS motifs, and the POSSUM program (http://zlab.bu.edu/~mfrith/possum/).

We also used a public thyroid microarray normalised data set to test the differential expression of *DAP3* and its best candidate regulator genes in ten FTA, seven OTA, eight OTC, fifty-one PTC and four NT (Giordano *et al*, 2006). The log-expression values of duplicate genes were averaged and the antilogarithm values were used for plotting as presented by the authors. Significance of difference between the tumour types and the NT tissues was assessed by using Wilcoxon tests at 5% risk.

### Quantitative PCR

Quantification analyses were carried out using the Chromo4 Real Time PCR Detector technology (Bio-Rad, Hercules, CA, USA) and the iQ SYBR Green Supermix following the manufacturer's instructions (Bio-Rad). The standards were obtained by PCR carried out on total cDNA of NT tissue as described elsewhere (Jacques *et al*, 2006). The forward and reverse primers used were as follow: for *DAP3*, 5′-GCTGGGAAAGGAAGGATTTG-3′ and 5′-TTCGCGTTACTTAGGAACAG-3′ (Tm: 57 °C); for *ESRRA*, 5′-AAGACAGCAGCCCCAGTGAA-3′ and 5′-ACACCCAGCACCAGCACCT-3′ (Tm: 64 °C); for *ELK1*, 5′-GGCTACGCAAGAACAAGACC-3′ and 5′-TTTGGCATGGTGGAGGTAAC-3′ (Tm: 61 °C); for *RUNX1*, 5′-TGTGATGGCTGGCAATGATG-3′ and 5′-GCCCATCCACTGTGATTTTG-3′ (Tm: 60 °C); for actin, 5′-CGACATGGAGAAAATCTGGC-3′ and 5′-AGGTCCAGACGCAGGATGG-3′ (57 °C). Gene expression for *DAP3*, *ESRRA*, *ELK1* and *RUNX1* was normalised by reference to the actin gene expression for each sample.

### Immunohistochemistry

Tumour samples (100) and normal conterparts (61) were used for tissue array construction, where each sample were represented by three spots (0.6 mm diameter); immunostaining was carried out using the standard avidin–biotin peroxidase technique as described earlier (Kononen *et al*, 1998; Jacques *et al*, 2005). The primary antibody was either a monoclonal anti-DAP3 (Transduction Laboratories, Lexington, UK; 1/50) or a monoclonal antibody against a mitochondrial subunit of the respiratory chain complex IV (clone 113-1, Biogenex laboratories, Inc., San Ramon, CA, USA; dilution 1/50). Diaminobenzidin was used as the chromogen and haematoxylin as the nuclear counterstain. Negative controls were carried out by replacing the primary antibody with buffer. The analysis takes into account the intensity of the signal and the percentage of positive cells scored as 0 (no staining), 1 (<25%), 2 (25–75%) and 3 (75% <). Overrepresentation of positive cells in tumours compared with paired normal tissues was assessed by the two-tailed Fisher's exact test (*P*⩽0.05). The correlation between DAP3 expression and the mitochondrial antigen expression was assessed by the Spearman's test.

## Results

### Characterisation of *DAP3-*related genes

Using the TMM web server and a meta-analysis of 100 public microarray data sets, we found 337 genes significantly correlated with *DAP3* (Supplementary Table 1). This gene collection included more mitoribosomal genes (six genes, e.g., *MRPL9*, *MRPL16*, *MRPL3*, *MRPS18B*, *MRPS31* and *MRPS17*) than expected by chance when considering 39 949 human genes from NCBI Gene database (Fisher's exact test, *P*<1.31E-04). To avoid redundancy in further analyses, we included only the 331 non-mitoribosomal genes in this gene set. Two other sets represent the large (44 out of 52 genes) and the small (27 out of 33 genes) mitoribosomal subunits, respectively.

We analysed the gene promoter sequences for enrichment in TFBS motifs in each set separately (Table 1). We found nine transcriptional factors with significantly enriched motifs, some motifs being common to gene sets. Transcription factor binding site motifs for the two transcriptional activators of mitochondrial biogenesis NRF-1 and GABPA were found in all the gene sets. Transcription factor binding site motifs for ELK1 and ELK4 were overrepresented in the 69 out of 331 non-mitoribosomal genes and the mitoribosomal large subunit genes. These two proteins are members of the Ets family of transcription factors and of the ternary complex factor (TCF) subfamily proteins, which act in the promoter of immediate early-class genes such as the *c-fos* proto-oncogene (Yang *et al*, 2003). Transcription factor binding site motifs for RUNX1 and HOX11-CTF1 were specific to the set of non-mitoribosomal genes. The *RUNX1* gene encodes an important regulator of haematopoiesis and is the target of genetic alterations during leukaemogenesis (Mikhail *et al*, 2006). The upregulation and interaction of HOX11 and CTF1 is implicated in the immortalisation of haematopoietic precursor cells (Zhang *et al*, 1999). Transcription factor binding site motifs for PPARG-RXRA were specific to the mitoribosomal large subunit set. PPARG and RXRA are respectively implicated in adipocyte differentiation and lipogenesis (Metzger *et al*, 2005). Transcription factor binding site motifs for ESRRA and TAL1 were specific to the small mitoribosomal subunit set. Estrogen-related receptor alpha is a regulator of *β*-oxidation implicated in PGC-1*α*-induced mitochondrial biogenesis (Scarpulla, 2006). TAL1 is required for normal haematopoiesis; its aberrant expression leads to T-cell acute lymphoblastic leukaemia (Palomero *et al*, 2006).

We analysed the *DAP3* gene promoter with the POSSUM program and the collection of 125 TFBS motifs. The motifs showing the highest-scoring TFBS detached from the background concerned the nine transcriptional factors (or combinations of transcriptional factors) extracted earlier with the analysis of gene promoters. These best-scoring sites, depicted in Table 2, were distributed in the promoter region covering −736 to +946 on both strands.

### mRNA expression of DAP3 and associated transcription factors

**Figure 2:**
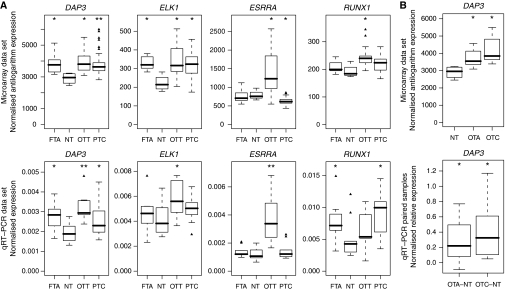
(**A**) Differential expression of *DAP3*, *ELK1*, *ESRRA* and *RUNX1* in tumours and normal thyroid (NT) samples. We used a public microarray data set (Giordano *et al*, 2006) containing data for 10 follicular thyroid adenomas (FTA), four NT, 15 oncocytic thyroid tumours (OTT) and 51 papillary thyroid carcinomas (PTC). We considered differential expression with two-sided Wilcoxon test (^*^ for *P*<0.05; ^**^ for *P*<0.005). The box-plot representation shows the median value of mRNA expression (bold line), the lower and upper limits of each box representing the first and third quartiles, respectively. Whiskers represent the limits of extreme measurements. *P*-values are shown in brackets. A set of 40 thyroid samples (10 FTA, 10 NT, 10 OTT and 10 PTC) was analysed by quantitative PCR (qPCR). Each expression level is normalised with respect to the normal tissue, the unit value representing isoexpression. We considered overexpression with one-sided Wilcoxon test (^*^ for *P*<0.05; ^**^ for *P*<0.005). (**B**) *DAP3* mRNA expression levels in oncocytic thyroid adenomas (OTA) and oncocytic thyroid carcinomas (OTC). A set of twelve OTA and five OTC samples paired with normal tissue was analysed by qPCR (bottom plot). *DAP3* expression levels in tumours are subtracted by paired normal levels. *DAP3* gene expression is shown in seven OTA, eight OTC and four NT samples from the microarray data set (top plot). We considered overexpression with one-sided Wilcoxon test (^*^ for *P*<0.05). The box-plot representations for microarray and qPCR results are as in A.

A publicly available thyroid microarray data set (Giordano *et al*, 2006) showed overexpression of *DAP3*, *ESRRA*, *ELK1* and *RUNX1* genes in 15 OTT samples when compared with four NT samples (*P*<0.05, two-sided Wilcoxon test; Figure 2A). Death-associated protein 3 and ELK1 were also differentially expressed in 10 FTA and 51 PTC samples, and ESRRA in PTC.

Overexpression of these four genes was further evaluated by qPCR in 10 OTT, 10 PTC, 10 FTA and 10 control tissue samples (Figure 2A). *DAP 3*, *ELK1* and *ESRRA* were confirmed to be significantly overexpressed in OTT (*P*<0.05, one-sided Wilcoxon test). Death-associated protein 3 overexpression in FTA and PTC was also validated (*P*<0.05). For *RUNX1*, the gene expression was not significantly changed for the OTT but significant differences were shown for the FTA and the PTC (*P*<0.05).

The *DAP3* gene expression was also explored in an independent qPCR analysis with 12 OTA and five OTC samples paired with unaffected surrounding tissue and compared with the seven OTA and eight OTC samples from the microarray data set (Figure 2B). The *DAP3* overexpression was confirmed in both data sets for OTA and OTC compared with the NT tissue (*P*<0.05, one-sided Wilcoxon test).

### Immunohistochemistry

**Figure 3:**
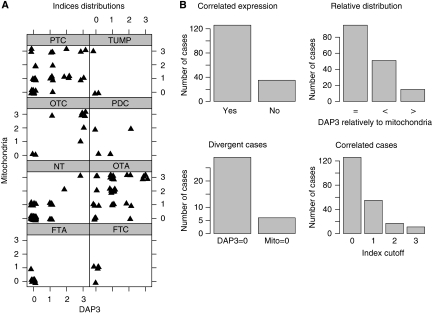
Tissue array were constructed with 100 thyroid tumours and 61 normal thyroid samples. Immunohistochemistry analysis was carried out using the standard avidin–biotin peroxidase technique with either a monoclonal anti-DAP3 or a mitochondrial antigen antibody as primary antibodies. Antigen expression is coded from 0 (no staining) to three (strong staining). (**A**) Distribution of the number of positive cells for DAP3 and the mitochondrial antigen analysed. Considering the distribution of the expression values of DAP3 and the mitochondrial antigen in the different types of tumours, the correlation between the two proteins expression assessed by the Spearman's test shows a significant and positive correlation (*ρ*=0.65, *P*<0.01). (**B**) Statistical analysis of the expression correlation between DAP3 and a mitochondrial antigen in tumours. Statistical analysis of the distribution of the values is represented in histograms. Expression values of the two antigens were considered correlated when they were both non-null and both equal to zero (correlated expression). Divergence was defined when one antigen showed a non-null expression, whereas the other antigen was not expressed (divergent cases). The relative distribution of the DAP3 antigen expression is shown when it is equal (=), lower (<) or greater (>) than the mitochondrial antigen (relative distribution). The number of correlated cases is shown for several expression value cutoffs (correlated cases).

The DAP3 expression was negative for most of the NT samples (52 out of 61), poorly differentiated carcinomas (two out of four; not significant), FTC (five out of five), TUMP (three out of three) and FTA (eight out of eight). A significant overexpression of DAP3 was detected in OTC (seven out of nine), OTA (26 out of 37) and PTC (16 out of 34) compared with normal tissue (*P*<0.05). The distribution of percentage of positive cells for DAP3 and for the mitochondrial antigen in the 161 samples is represented in Figure 3A. The analysis of correlated expression, relative distribution, divergent cases and correlated cases are shown in Figure 3B. The Spearman's test gave a positive and significant correlation between the expression of the mitochondrial antigen and the expression of DAP3 (*ρ*=0.65, *P*<0.01). The DAP3 staining and the mitochondrial antigen staining were both localised in the cytoplasm with a granular aspect, sometimes more intense close to the nucleus. In PTC, the staining was sometimes concentrated in both apical and basal region (data not shown).

## Discussion

One of the main functions ascribed to human DAP3 concerns its implication in the extrinsic pathway of apoptotis (Kissil *et al*, 1995; Mukamel and Kimchi, 2004). However, the suppression of DAP3 *in vivo* has also shown its role in the efficacy of mitochondrial translation and respiration (Kim *et al*, 2007).

The comparison of the promoter sequence of the *DAP3* gene with that of other genes could be expected to contribute to the exploration of the mitoribosomal function of DAP3. The analysis of gene correlations in 100 microarray data sets showed 337 genes potentially coexpressed with the *DAP3* gene (Supplementary Table 1). These genes were mainly related to nucleic acid metabolism, transcription and RNA processing, protein metabolism and mitochondrial function (data not shown). Using 125 TFBS position–weight matrices, we found six transcriptional factors potentially involved in the regulation of the 69 out of 331 non-mitochondrial *DAP3* coexpressed genes. The analysis of the two mitoribosomal subunits proteins showed three supplementary transcriptional factors related to the potential regulation of the majority of both large and small mitoribosomal constituents (Table 1). These factors are known to be implicated in mitochondrial biogenesis (GABPA, NRF-1, PPARG-RXRA and ESRRA) or in tumourigenesis (ELK1, ELK4, HOX11-CTF1, RUNX1 and TAL1-TCF3). The results obtained using here a highly selective process, are reinforced by the overexpression of *ELK1*, *RUNX1* and *ESRRA*, observed in oncocytic tumours described by Giordano *et al*. (Giordano *et al*, 2006) (Figure 2A).

Screening the *human death-associated protein 3* (*hDAP3*) gene promoter with the 125 TFBS motifs, these nine transcriptional factors presented the highest-scoring sites (Table 2). Then *hDAP3* may be associated with tumourigenesis and mitochondrial biogenesis through a mechanism of coregulation at the transcriptional level. Our *in silico* findings are compatible with the published reports concerning the implication of *hDAP3* in tumourigenesis or aggressive cellular processes and in the efficacy of mitochondrial translation (Mariani *et al*, 2001; Sasaki *et al*, 2004). However, the mechanism of *DAP3* gene regulation needs further experimental investigation.

In NT tissue, apoptotic cell death is a rare event; the apoptotic process is blocked at the step of activation of pro-caspase 3 (Mezosi *et al*, 2005). In the thyroid carcinomas, the induction of apoptosis by the Fas ligand is stopped before the activation of caspase 8 (Mitsiades *et al*, 2000). In our laboratory, a microarray analysis of 93 follicular thyroid tumours showed significant downregulation of caspase 3 expression in the 34 oncocytic tumours studied (data not shown) (Fontaine *et al*, 2008).

In this publication, we show a significant overexpression of *DAP3* mRNA in three types of thyroid tumour (FTA, PTC and OTT) compared with their NT tissue counterparts (Figure 2A). The IHC staining of the DAP3 protein was similar to the one observed for a mitochondrial antigen, suggesting that the major pool of DAP3 is localised in the mitochondrion. The number of samples used here in each type of tumours avoids trying to subdivide the tumour types on the basis of DAP3 expression, as this would reduce drastically the power of the statistical analysis for subtypes. Rather, searching for a link between the expression of DAP3 and the mitochondrial biogenesis, we show that, when thyroid tumours have a rich mitochondrial content, whether they belong to the oxyphilic tumour categories, to the papillary carcinomas or UMP type, DAP3 overexpression is dependent on the cell mitochondrial content (Figure 3). These data suggest that DAP3 may be necessary for an increased mitochondrial biogenesis.

In an earlier microarray analysis carried out on thyroid tumours, we found that oncocytomas overexpress 126 genes compared with other tumours. Among these 126 genes, the *Mitochondrial Ribosomal Protein Large subunit 49* (*MRPL49*) and 13 genes coding for subunits of the OXPHOS complexes were represented (Baris *et al*, 2004). A two-step study on the basis of differential display and macroarray analysis of six oncocytic thyroid adenoma samples showed that 12 of the 30 genes upregulated at least two-fold in the tumours were mtDNA-encoded genes (Jacques *et al*, 2005). Similarly to the data in renal and salivary oncocytomas, these analyses on thyroid oncocytoma suggest a coordinated regulation of the nuclear and mtDNA genes (Heddi *et al*, 1996; Baris *et al*, 2005). Functional analyses of the thyroid oncocytic tumours also showed that overrepresented OXPHOS complexes are functional (Savagner *et al*, 2001a, 2001b). These findings are in favour of efficacious mitochondrial translation in these tumours. In the thyroid oncocytoma, the upregulation of hDAP3 is also associated with low apoptosis. These tumours may then be a good model to study specifically the function of DAP3 in the efficacy or fidelity of mitochondrial translation.

The results of our study taken together with the implication of DAP3 in the composition of the small subunit of the mitochondrial ribosome, suggest that the protein may serve as an actor or regulator of mitochondrial protein synthesis. DAP3 could thus play a role in maintaining mitochondrial homoeostasis on one hand, and participate in the process of tumourigenesis on the other. Furthermore, if the tumourigenetic process calls for increased mitochondrial biogenesis, a defect in even one of the proteins of the mitochondrial ribosome, such as DAP3, could lead to cell apoptosis.

## Figures and Tables

**Table 1:** Transcription factors involved in the regulation of *DAP3*-corelated genes, and MRP genes of the large and small subunits of the mitoribosome

				***P*-values**	
**Genes set**	**Motif name**	**Motif family**	**Raw score**	**Promoters**	**Cpg**
331 potential DAP3 correlated genes	GABPA	ETS	4.63	0.002	0
	ELK1	ETS	1.37	0	0.012
	NRF1		1.65	0.011	0.001
	ELK4	ETS	−1.31	0	0
	RUNX1	RUNT	−2.6	0.028	0.044
	Hox11-CTF1	HOMEO/CAAT	−2.97	0.031	0.02
Mitochondrial large subunit genes	NRF1		9.91	0	0
	ELK4	ETS	2.97	0	0
	GABPA	ETS	2.88	0.001	0.001
	ELK1	ETS	1.99	0	0.005
	PPARG-RXRA	Nuclear receptor	0.598	0.042	0.003
Mitochondrial small subunit genes	ESRRA		3.29	0	0.021
	NRF1		3.14	0.002	0
	TAL1-TCF3	bHLH	2.25	0	0.006
	GABPA	ETS	1.82	0.022	0.012

The exploration of the promoter region (−1000 to +1000 starting from the TSS) of 69 out of 331 non-mitoribosomal *DAP3*-related genes, 44 out of 52 MRP large subunit genes and 27 out of 33 MRP small subunit genes, including *DAP3* is recapitulated here. The name of the motif, when possible, the family, together with the raw score and the *P*-value computed by the Clover program are indicated for each best-scoring sites presented. The relative frequencies of the motifs are compared with two background models, a set of 6461 human gene promoters (−1000 to +1000 from the TSS) and a set of 27 555 CpG island sequences.

**Table 2:** Potential target-binding sites for regulators in the *DAP3* gene promoter

**Motif**	**Sequence**	**Start position**	**End position**	**Strand**	**Score**
ELK1	CTTACGGAAA	−458	−449	+	5.92
	TTTCCCCTCG	345	354	−	5.51
	TGTCAGGGAC	−79	−70	−	5.49
ELK4	TCCGGGAGT	−93	−85	+	4.87
	ACTGGAATT	938	946	+	4.64
	GTTTCCCCT	344	352	−	4.17
ERR-*α*	TGTCCTCGA	−232	−224	−	7.48
	AGACCTTGT	−270	−262	−	6.41
	TGACATTTA	−345	−337	−	5.8
	TCAGGGACA	−77	−69	+	5.02
GABPA	AGAGGAAGGG	898	907	+	7.18
	CTCCTCCGCT	185	194	−	6.99
	CCCTCCCGCT	215	224	−	6.56
	CGCGGCAGGG	−287	−278	+	6.44
Hox11-CTF1	AGGGAGGGAGCTAA	418	431	+	3.27
	TTTCCCCTCGCAAA	345	358	+	3.11
NRF1	CGCGCGTGCGCC	833	844	+	5.57
	CGCGCGTGCGCC	833	844	−	5.49
	AGCGCAGGCCCT	690	701	−	4.75
RUNX1	GACCACAAC	−679	−671	−	6.05
	CACCACAGA	−276	−268	−	4.74
TAL1-TCF3	AGACCATCTGTC	−399	−388	+	9.93
	ACCATCTGTCTG	−397	−386	−	7.18
PPARG-RXRA	TTTTGGCCCTTCACATTTAC	−736	−717	−	2.6

The POSSUM program and 125 TFBS motifs were used to analyse the *DAP3* gene promoter region (−1000 to +1000 nucleotides around the TSS). The table shows the sequence, start and end positions of the motifs, the strand on the chromosome and the computed score for the highest-scoring TFBS motifs.
