# Supplementary material for: Death-associated protein 3 is overexpressed in human thyroid oncocytic tumours
Source: Br J Cancer. 2009 Jun 16;101(1):132–8. doi: 10.1038/sj.bjc.6605111 (PMC2713694; doi:10.1038/sj.bjc.6605111)
Supplement: Supplementary Table 1 [file 6605111x1.doc]

**Supplemental Table 1: list of the 337 potential *DAP3* co-regulated genes.**

| *Symbol* | *Name* | *#ds* | *datasets* | *score* |
| --- | --- | --- | --- | --- |
| CCT3 | Chaperonin containing TCP1, subunit 3 (gamma) | 9 | GSE1133, armstrong-mll, su-human, vantveer-breast, hedenfalk-breast, butte-relevance, GDS531, staunton-nci60, diehn-cd28 | 0.76 |
| PSMB4 | Proteasome (prosome, macropain) subunit, beta type, 4 | 9 | khatua-astrocytoma, shipp-dlbcl, GSE1133, armstrong-mll, yeoh-leukemia, GDS531, huang-breast, diehn-cd28, welsh-prostate | 0.73 |
| EPRS | Glutamyl-prolyl-tRNA synthetase | 9 | shipp-dlbcl, GSE1133, armstrong-mll, yeoh-leukemia, su-human, huang-breast, leung-gastric, GDS531, diehn-cd28 | 0.69 |
| SDHC | Succinate dehydrogenase complex, subunit C, integral membrane protein, 15kDa | 8 | GDS558, sorlie-breasttumor, vantveer-breast, leung-gastric, GDS531, staunton-nci60, GDS462, diehn-cd28 | 0.72 |
| SSBP1 | Single-stranded DNA binding protein 1 | 7 | shipp-dlbcl, GDS558, GSE1133, ross-nci60, su-human, GDS462, diehn-cd28 | 0.79 |
| SNRPE |  | 7 | GSE1133, leung-gastric, ross-nci60, yeoh-leukemia, GDS531, su-human, diehn-cd28 | 0.71 |
| KARS | Lysyl-tRNA synthetase | 6 | GDS511, GSE1133, su-human, diehn-cd28, GDS462, GDS563 | 0.88 |
| PSMA6 | Proteasome (prosome, macropain) subunit, alpha type, 6 | 6 | GDS558, GSE1133, whitfield-cellcycle, su-human, diehn-cd28, GDS462 | 0.83 |
| GA17 |  | 6 | GSE1133, leung-gastric, whitfield-cellcycle, su-human, diehn-cd28, GDS462 | 0.81 |
| APEX1 | APEX nuclease (multifunctional DNA repair enzyme) 1 | 6 | GDS511, leung-gastric, GSE1133, luo-prostate, diehn-cd28, welsh-prostate | 0.77 |
| PSMD4 | Proteasome (prosome, macropain) 26S subunit, non-ATPase, 4 | 6 | shipp-dlbcl, GDS558, leung-gastric, GSE1133, staunton-nci60, diehn-cd28 | 0.76 |
| CCT6A | Chaperonin containing TCP1, subunit 6A (zeta 1) | 6 | GSE1133, khan-bluecell, armstrong-mll, luo-prostate, su-human, diehn-cd28 | 0.75 |
| XRCC5 | X-ray repair complementing defective repair in Chinese hamster cells 5 (double-strand-break rejoining; Ku autoantigen, 80kDa) | 6 | GSE1133, leung-gastric, GDS651, huang-breast, GDS462, diehn-cd28 | 0.75 |
| PA2G4 |  | 6 | sarwal-allograft, GDS511, GSE1133, leung-gastric, ross-nci60, diehn-cd28 | 0.74 |
| CBX3 | Chromobox homolog 3 (HP1 gamma homolog, Drosophila) | 6 | GSE1133, leung-gastric, ross-nci60, whitfield-cellcycle, su-human, diehn-cd28 | 0.7 |
| PSMA1 | Proteasome (prosome, macropain) subunit, alpha type, 1 | 5 | GSE1133, GDS564, su-human, diehn-cd28, GDS462 | 0.85 |
| PSMB1 | Proteasome (prosome, macropain) subunit, beta type, 1 | 5 | GDS511, GSE1133, su-human, macdonald-mblastoma, diehn-cd28 | 0.84 |
| UQCRC2 | Ubiquinol-cytochrome c reductase core protein II | 5 | GSE1133, su-human, diehn-cd28, GDS462, welsh-prostate | 0.83 |
| BTF3 | Basic transcription factor 3 | 5 | GDS558, cheok-leukemia, GSE1133, su-human, diehn-cd28 | 0.81 |
| PSMA5 | Proteasome (prosome, macropain) subunit, alpha type, 5 | 5 | GDS558, GSE1133, su-human, rickman-glioma, diehn-cd28 | 0.81 |
| RABGGTB | Rab geranylgeranyltransferase, beta subunit | 5 | GDS511, GSE1133, macdonald-mblastoma, su-human, diehn-cd28 | 0.81 |
| AATF | Apoptosis antagonizing transcription factor | 5 | GDS511, GSE1133, su-human, diehn-cd28, GDS563 | 0.8 |
| PSMD14 | Proteasome (prosome, macropain) 26S subunit, non-ATPase, 14 | 5 | GSE1133, su-human, dyrskjot-bldderstage, diehn-cd28, GDS462 | 0.8 |
| TARS | Threonyl-tRNA synthetase | 5 | GSE1133, su-human, diehn-cd28, GDS462, welsh-prostate | 0.8 |
| CCT2 | Chaperonin containing TCP1, subunit 2 (beta) | 5 | cheok-leukemia, GSE1133, GDS564, su-human, diehn-cd28 | 0.78 |
| CLNS1A | Chloride channel, nucleotide-sensitive, 1A | 5 | leung-gastric, GSE1133, su-human, diehn-cd28, GDS462 | 0.78 |
| CBFB | Core-binding factor, beta subunit | 5 | GDS511, GSE1133, leung-gastric, su-human, diehn-cd28 | 0.77 |
| COPS5 | COP9 constitutive photomorphogenic homolog subunit 5 (Arabidopsis) | 5 | sarwal-allograft, GSE1133, armstrong-mll, whitfield-cellcycle, diehn-cd28 | 0.77 |
| COX7B | Cytochrome c oxidase subunit VIIb | 5 | GSE1133, su-human, staunton-nci60, diehn-cd28, GDS462 | 0.77 |
| EIF3S1 | Eukaryotic translation initiation factor 3, subunit 1 alpha, 35kDa | 5 | sarwal-allograft, leung-gastric, cheung-lymphoblastoid, diehn-cd28, GDS462 | 0.77 |
| KIAA0117 |  | 5 | khatua-astrocytoma, GSE1133, GDS531, su-human, diehn-cd28 | 0.77 |
| PWP1 | PWP1 homolog (S. cerevisiae) | 5 | sarwal-allograft, GSE1133, golub-leukemia, cheung-lymphoblastoid, diehn-cd28 | 0.77 |
| PSMA3 | Proteasome (prosome, macropain) subunit, alpha type, 3 | 5 | sarwal-allograft, GSE1133, su-human, macdonald-mblastoma, diehn-cd28 | 0.75 |
| DKC1 | Dyskeratosis congenita 1, dyskerin | 5 | GSE1133, leung-gastric, ross-nci60, su-human, diehn-cd28 | 0.74 |
| NAP1L1 |  | 5 | GSE1133, yeoh-leukemia, macdonald-mblastoma, su-human, diehn-cd28 | 0.74 |
| SNRPF | Small nuclear ribonucleoprotein polypeptide F | 5 | shipp-dlbcl, GSE1133, leung-gastric, su-human, diehn-cd28 | 0.74 |
| CCT4 | Chaperonin containing TCP1, subunit 4 (delta) | 5 | GDS534, GSE1133, leung-gastric, su-human, diehn-cd28 | 0.71 |
| DDX21 | DEAD (Asp-Glu-Ala-Asp) box polypeptide 21 | 5 | GSE1133, leung-gastric, ross-nci60, su-human, diehn-cd28 | 0.71 |
| EIF3S7 | Eukaryotic translation initiation factor 3, subunit 7 zeta, 66/67kDa | 5 | cheok-leukemia, yeoh-leukemia, su-human, chang-fibroblasts, diehn-cd28 | 0.71 |
| SHFM1 | Split hand/foot malformation (ectrodactyly) type 1 | 5 | shipp-dlbcl, ross-nci60, armstrong-mll, su-human, diehn-cd28 | 0.71 |
| SCAMP3 | Secretory carrier membrane protein 3 | 5 | leung-gastric, GDS531, sorlie-breasttumor, vantveer-breast, diehn-cd28 | 0.7 |
| GSPT1 | G1 to S phase transition 1 | 5 | leung-gastric, GDS531, su-human, rickman-glioma, diehn-cd28 | 0.69 |
| NICE-4 |  | 5 | GSE1133, leung-gastric, GDS531, huang-breast, diehn-cd28 | 0.68 |
| RFC4 | Replication factor C (activator 1) 4, 37kDa | 5 | sarwal-allograft, leung-gastric, ross-nci60, su-human, diehn-cd28 | 0.67 |
| FLJ10326 |  | 5 | leung-gastric, ross-nci60, armstrong-mll, GDS531, huang-breast | 0.66 |
| PSMA2 | Proteasome (prosome, macropain) subunit, alpha type, 2 | 4 | GDS558, su-human, GDS462, diehn-cd28 | 0.86 |
| DKFZP564M182 | | 4 | GDS511, su-human, diehn-cd28, GDS462 | 0.84 |
| POLR2B | Polymerase (RNA) II (DNA directed) polypeptide B, 140kDa | 4 | khatua-astrocytoma, GSE1133, GDS564, diehn-cd28 | 0.83 |
| UQCRH | Ubiquinol-cytochrome c reductase hinge protein | 4 | GSE1133, su-human, GDS462, welsh-prostate | 0.83 |
| MCTS1 | Malignant T cell amplified sequence 1 | 4 | GSE1133, luo-prostate, diehn-cd28, GDS462 | 0.82 |
| POLR2F | Polymerase (RNA) II (DNA directed) polypeptide F | 4 | khatua-astrocytoma, shipp-dlbcl, luo-prostate, diehn-cd28 | 0.82 |
| SET | SET translocation (myeloid leukemia-associated) | 4 | GSE1133, luo-prostate, diehn-cd28, GDS462 | 0.81 |
| C1QBP | Complement component 1, q subcomponent binding protein | 4 | sarwal-allograft, su-human, diehn-cd28, GDS462 | 0.79 |
| COPS3 | COP9 constitutive photomorphogenic homolog subunit 3 (Arabidopsis) | 4 | GSE1133, su-human, diehn-cd28, GDS462 | 0.79 |
| PPP2R5E | Protein phosphatase 2, regulatory subunit B (B56), epsilon isoform | 4 | GSE1133, leung-gastric, cheung-lymphoblastoid, diehn-cd28 | 0.79 |
| PSMA4 | Proteasome (prosome, macropain) subunit, alpha type, 4 | 4 | GSE1133, armstrong-mll, su-human, diehn-cd28 | 0.79 |
| PSMC1 | Proteasome (prosome, macropain) 26S subunit, ATPase, 1 | 4 | GDS558, GSE1133, leung-gastric, chaussabel-parasite | 0.79 |
| AK074970 |  | 4 | leung-gastric, GSE1133, su-human, diehn-cd28 | 0.78 |
| COX7A2L | Cytochrome c oxidase subunit VIIa polypeptide 2 like | 4 | GDS558, cheok-leukemia, GSE1133, leung-gastric | 0.78 |
| EIF4A1 | Eukaryotic translation initiation factor 4A, isoform 1 | 4 | leung-gastric, GSE1133, su-human, luo-prostate | 0.78 |
| ETFA | Electron-transfer-flavoprotein, alpha polypeptide (glutaric aciduria II) | 4 | GSE1133, su-human, GDS462, diehn-cd28 | 0.78 |
| H2AFY |  | 4 | GSE1133, GDS564, diehn-cd28, welsh-prostate | 0.78 |
| SFRS2 | Splicing factor, arginine/serine-rich 2 | 4 | leung-gastric, GSE1133, su-human, diehn-cd28 | 0.78 |
| COX7C | Cytochrome c oxidase subunit VIIc | 4 | GSE1133, staunton-nci60, GDS462, butte-relevance | 0.77 |
| GART | Phosphoribosylglycinamide formyltransferase, phosphoribosylglycinamide synthetase, phosphoribosylaminoimidazole synthetase | 4 | leung-gastric, su-human, cheung-lymphoblastoid, diehn-cd28 | 0.77 |
| HSPA4 | Heat shock 70kDa protein 4 | 4 | GSE1133, leung-gastric, su-human, diehn-cd28 | 0.77 |
| M11S1 |  | 4 | GSE1133, leung-gastric, diehn-cd28, GDS462 | 0.77 |
| PTD004 |  | 4 | leung-gastric, GSE1133, diehn-cd28, GDS462 | 0.77 |
| VBP1 | Von Hippel-Lindau binding protein 1 | 4 | GSE1133, leung-gastric, diehn-cd28, GDS462 | 0.77 |
| CCT7 | Chaperonin containing TCP1, subunit 7 (eta) | 4 | leung-gastric, GDS564, su-human, cheung-lymphoblastoid | 0.76 |
| KIAA0179 | KIAA0179 | 4 | leung-gastric, GSE1133, su-human, diehn-cd28 | 0.76 |
| NME1 | Non-metastatic cells 1, protein (NM23A) expressed in | 4 | su-human, macdonald-mblastoma, diehn-cd28, butte-relevance | 0.76 |
| RBMX |  | 4 | leung-gastric, GSE1133, su-human, diehn-cd28 | 0.76 |
| TFAM | Transcription factor A, mitochondrial | 4 | GSE1133, leung-gastric, rinaldi-leukemia, diehn-cd28 | 0.76 |
| TRIP15 |  | 4 | GSE1133, whitfield-cellcycle, su-human, diehn-cd28 | 0.76 |
| DDX18 | DEAD (Asp-Glu-Ala-Asp) box polypeptide 18 | 4 | leung-gastric, GSE1133, ross-nci60, diehn-cd28 | 0.75 |
| FNBP3 |  | 4 | leung-gastric, GSE1133, su-human, diehn-cd28 | 0.75 |
| FUSIP1 |  | 4 | GSE1133, whitfield-cellcycle, su-human, diehn-cd28 | 0.75 |
| PPP1CC | Protein phosphatase 1, catalytic subunit, gamma isoform | 4 | GSE1133, leung-gastric, su-human, diehn-cd28 | 0.75 |
| SNRPA1 | Small nuclear ribonucleoprotein polypeptide A' | 4 | leung-gastric, GSE1133, su-human, diehn-cd28 | 0.75 |
| SUMO1 | SMT3 suppressor of mif two 3 homolog 1 (S. cerevisiae) | 4 | leung-gastric, GSE1133, su-human, diehn-cd28 | 0.75 |
| TCEA1 |  | 4 | leung-gastric, GSE1133, su-human, diehn-cd28 | 0.75 |
| TRAP1 | TNF receptor-associated protein 1 | 4 | leung-gastric, macdonald-mblastoma, diehn-cd28, welsh-prostate | 0.75 |
| CCT5 | Chaperonin containing TCP1, subunit 5 (epsilon) | 4 | GSE1133, leung-gastric, su-human, diehn-cd28 | 0.74 |
| EIF2S3 | Eukaryotic translation initiation factor 2, subunit 3 gamma, 52kDa | 4 | GSE1133, leung-gastric, su-human, diehn-cd28 | 0.74 |
| MAPRE1 | Microtubule-associated protein, RP/EB family, member 1 | 4 | GSE1133, leung-gastric, su-human, diehn-cd28 | 0.74 |
| PRDX3 | Peroxiredoxin 3 | 4 | GSE1133, leung-gastric, diehn-cd28, GDS462 | 0.74 |
| GDI2 | GDP dissociation inhibitor 2 | 4 | leung-gastric, armstrong-mll, su-human, diehn-cd28 | 0.73 |
| MTIF2 | Mitochondrial translational initiation factor 2 | 4 | GSE1133, leung-gastric, su-human, diehn-cd28 | 0.73 |
| RBBP4 | Retinoblastoma binding protein 4 | 4 | leung-gastric, GSE1133, diehn-cd28, welsh-prostate | 0.73 |
| SFRS10 | Splicing factor, arginine/serine-rich 10 (transformer 2 homolog, Drosophila) | 4 | leung-gastric, GSE1133, whitfield-cellcycle, diehn-cd28 | 0.73 |
| SNRPD3 | Small nuclear ribonucleoprotein D3 polypeptide 18kDa | 4 | GSE1133, leung-gastric, dyrskjot-bldderstage, diehn-cd28 | 0.73 |
| SRPK1 | SFRS protein kinase 1 | 4 | leung-gastric, whitfield-cellcycle, su-human, diehn-cd28 | 0.73 |
| YARS | Tyrosyl-tRNA synthetase | 4 | leung-gastric, GSE1133, su-human, diehn-cd28 | 0.73 |
| GNPAT | Glyceronephosphate O-acyltransferase | 4 | GSE1133, khan-bluecell, GDS531, GDS462 | 0.72 |
| IMMT | Inner membrane protein, mitochondrial (mitofilin) | 4 | ross-nci60, whitfield-cellcycle, su-human, diehn-cd28 | 0.72 |
| MAK3 |  | 4 | leung-gastric, GSE1133, whitfield-cellcycle, diehn-cd28 | 0.72 |
| NPM1 | Nucleophosmin (nucleolar phosphoprotein B23, numatrin) | 4 | GSE1133, ross-nci60, su-human, diehn-cd28 | 0.72 |
| NSEP1 |  | 4 | GSE1133, leung-gastric, luo-prostate, diehn-cd28 | 0.72 |
| XPO1 | Exportin 1 (CRM1 homolog, yeast) | 4 | GSE1133, leung-gastric, whitfield-cellcycle, diehn-cd28 | 0.72 |
| ENSA |  | 4 | GSE1133, leung-gastric, GDS531, welsh-prostate | 0.71 |
| LRPPRC | Leucine-rich PPR-motif containing | 4 | GSE1133, leung-gastric, whitfield-cellcycle, diehn-cd28 | 0.71 |
| NASP | Nuclear autoantigenic sperm protein (histone-binding) | 4 | GSE1133, leung-gastric, su-human, diehn-cd28 | 0.71 |
| NUP153 |  | 4 | sarwal-allograft, GSE1133, leung-gastric, diehn-cd28 | 0.71 |
| PDCD2 |  | 4 | leung-gastric, GSE1133, su-human, diehn-cd28 | 0.71 |
| DEK | DEK oncogene (DNA binding) | 4 | GSE1133, leung-gastric, su-human, diehn-cd28 | 0.7 |
| DKFZP547E1010 | | 4 | GSE1133, leung-gastric, sorlie-breasttumor, diehn-cd28 | 0.7 |
| HNRPAB |  | 4 | GSE1133, ross-nci60, su-human, diehn-cd28 | 0.7 |
| PSMB7 | Proteasome (prosome, macropain) subunit, beta type, 7 | 4 | GSE1133, leung-gastric, luo-prostate, diehn-cd28 | 0.7 |
| SRP68 | Signal recognition particle 68kDa | 4 | sarwal-allograft, leung-gastric, whitfield-cellcycle, diehn-cd28 | 0.7 |
| USP10 | Ubiquitin specific peptidase 10 | 4 | leung-gastric, GSE1133, su-human, diehn-cd28 | 0.7 |
| ADAR | Adenosine deaminase, RNA-specific | 4 | GSE1133, leung-gastric, cheung-lymphoblastoid, huang-breast | 0.69 |
| CKS1B | CDC28 protein kinase regulatory subunit 1B | 4 | leung-gastric, su-human, cheung-lymphoblastoid, butte-relevance | 0.69 |
| METAP2 | Methionyl aminopeptidase 2 | 4 | leung-gastric, ross-nci60, whitfield-cellcycle, diehn-cd28 | 0.69 |
| MRPL9 | Mitochondrial ribosomal protein L9 | 4 | GSE1133, ross-nci60, GDS531, huang-breast | 0.69 |
| SSR2 | Signal sequence receptor, beta (translocon-associated protein beta) | 4 | shipp-dlbcl, GSE1133, staunton-nci60, vantveer-breast | 0.69 |
| XTP2 |  | 4 | leung-gastric, GSE1133, huang-breast, diehn-cd28 | 0.69 |
| H2AFV |  | 4 | GSE1133, leung-gastric, whitfield-cellcycle, su-human | 0.68 |
| SSB | Sjogren syndrome antigen B (autoantigen La) | 4 | GSE1133, leung-gastric, ross-nci60, su-human | 0.68 |
| HSPA9B |  | 4 | leung-gastric, ross-nci60, su-human, diehn-cd28 | 0.67 |
| TCFL1 |  | 4 | ross-nci60, GDS531, staunton-nci60, welsh-prostate | 0.64 |
| C14ORF166 | Chromosome 14 open reading frame 166 | 3 | GDS558, GSE1133, GDS462 | 0.92 |
| EIF3K |  | 3 | GSE1133, armstrong-mll, GDS462 | 0.88 |
| EIF3S3 | Eukaryotic translation initiation factor 3, subunit 3 gamma, 40kDa | 3 | GDS558, GSE1133, su-human | 0.88 |
| GTF3A | General transcription factor IIIA | 3 | GSE1133, su-human, GDS462 | 0.87 |
| AF271775 |  | 3 | khatua-astrocytoma, GSE1133, su-human | 0.86 |
| DRG1 | Developmentally regulated GTP binding protein 1 | 3 | GDS511, diehn-cd28, GDS462 | 0.86 |
| MRPS18B |  | 3 | khatua-astrocytoma, GSE1133, GDS462 | 0.86 |
| SOD1 | Superoxide dismutase 1, soluble (amyotrophic lateral sclerosis 1 (adult)) | 3 | shipp-dlbcl, diehn-cd28, GDS462 | 0.85 |
| APG12L |  | 3 | GSE1133, cheung-lymphoblastoid, diehn-cd28 | 0.83 |
| ATP5J | ATP synthase, H+ transporting, mitochondrial F0 complex, subunit F6 | 3 | GSE1133, diehn-cd28, GDS462 | 0.83 |
| C6ORF49 | Chromosome 6 open reading frame 49 | 3 | GSE1133, ross-nci60, GDS462 | 0.83 |
| COX5A | Cytochrome c oxidase subunit Va | 3 | diehn-cd28, GDS462, welsh-prostate | 0.83 |
| HNRPF | Heterogeneous nuclear ribonucleoprotein F | 3 | GSE1133, cheung-lymphoblastoid, diehn-cd28 | 0.83 |
| NDUFB5 | NADH dehydrogenase (ubiquinone) 1 beta subcomplex, 5, 16kDa | 3 | GSE1133, armstrong-mll, GDS462 | 0.83 |
| PPP6C | Protein phosphatase 6, catalytic subunit | 3 | sarwal-allograft, GSE1133, GDS462 | 0.83 |
| RPL4 |  | 3 | cheok-leukemia, GSE1133, su-human | 0.83 |
| TXNL1 | Thioredoxin-like 1 | 3 | luo-prostate, diehn-cd28, GDS462 | 0.83 |
| ESD | Esterase D/formylglutathione hydrolase | 3 | GSE1133, armstrong-mll, GDS462 | 0.82 |
| EXOSC8 | Exosome component 8 | 3 | GSE1133, su-human, diehn-cd28 | 0.82 |
| HSA9761 |  | 3 | GSE1133, rinaldi-leukemia, su-human | 0.82 |
| HSPC111 | Hypothetical protein HSPC111 | 3 | khatua-astrocytoma, GSE1133, diehn-cd28 | 0.82 |
| LOC91137 |  | 3 | GSE1133, diehn-cd28, GDS462 | 0.82 |
| POLD2 | Polymerase (DNA directed), delta 2, regulatory subunit 50kDa | 3 | diehn-cd28, GDS462, welsh-prostate | 0.82 |
| ATP6V0B | ATPase, H+ transporting, lysosomal 21kDa, V0 subunit b | 3 | cheung-lymphoblastoid, chang-fibroblasts, diehn-cd28 | 0.81 |
| CCND3 | Cyclin D3 | 3 | sarwal-allograft, cheung-lymphoblastoid, diehn-cd28 | 0.81 |
| CCT8 | Chaperonin containing TCP1, subunit 8 (theta) | 3 | GSE1133, su-human, diehn-cd28 | 0.81 |
| FBL | Fibrillarin | 3 | GSE1133, su-human, diehn-cd28 | 0.81 |
| HNRPC |  | 3 | GSE1133, su-human, diehn-cd28 | 0.81 |
| MFN2 | Mitofusin 2 | 3 | GDS478, GDS462, diehn-cd28 | 0.81 |
| MRPL16 | Mitochondrial ribosomal protein L16 | 3 | GSE1133, diehn-cd28, GDS462 | 0.81 |
| NDUFV2 | NADH dehydrogenase (ubiquinone) flavoprotein 2, 24kDa | 3 | GSE1133, diehn-cd28, GDS462 | 0.81 |
| NIPA2 | Non imprinted in Prader-Willi/Angelman syndrome 2 | 3 | GSE1133, diehn-cd28, hedenfalk-breast | 0.81 |
| PSMC4 | Proteasome (prosome, macropain) 26S subunit, ATPase, 4 | 3 | khatua-astrocytoma, GSE1133, diehn-cd28 | 0.81 |
| PSMC6 |  | 3 | GSE1133, macdonald-mblastoma, diehn-cd28 | 0.81 |
| SFRS3 | Splicing factor, arginine/serine-rich 3 | 3 | GSE1133, su-human, diehn-cd28 | 0.81 |
| UBE3A | Ubiquitin protein ligase E3A (human papilloma virus E6-associated protein, Angelman syndrome) | 3 | GDS558, GSE1133, diehn-cd28 | 0.81 |
| BCL2A1 | BCL2-related protein A1 | 3 | sarwal-allograft, cheung-lymphoblastoid, diehn-cd28 | 0.8 |
| COX4I1 | Cytochrome c oxidase subunit IV isoform 1 | 3 | cheok-leukemia, staunton-nci60, GDS462 | 0.8 |
| NCL | Nucleolin | 3 | GSE1133, su-human, diehn-cd28 | 0.8 |
| NSMAF | Neutral sphingomyelinase (N-SMase) activation associated factor | 3 | GSE1133, su-human, diehn-cd28 | 0.8 |
| PANK2 | Pantothenate kinase 2 (Hallervorden-Spatz syndrome) | 3 | leung-gastric, GSE1133, rinaldi-leukemia | 0.8 |
| SLBP | Stem-loop (histone) binding protein | 3 | GSE1133, GDS564, west-breast | 0.8 |
| SRRM1 | Serine/arginine repetitive matrix 1 | 3 | GSE1133, su-human, diehn-cd28 | 0.8 |
| TCERG1 | Transcription elongation regulator 1 | 3 | GSE1133, su-human, diehn-cd28 | 0.8 |
| UBE2N |  | 3 | GSE1133, cheung-lymphoblastoid, diehn-cd28 | 0.8 |
| ABCE1 | ATP-binding cassette, sub-family E (OABP), member 1 | 3 | GSE1133, luo-prostate, diehn-cd28 | 0.79 |
| API5 | Apoptosis inhibitor 5 | 3 | GSE1133, whitfield-cellcycle, diehn-cd28 | 0.79 |
| CSNK2A1 |  | 3 | GSE1133, diehn-cd28, GDS462 | 0.79 |
| CTPS | CTP synthase | 3 | leung-gastric, cheung-lymphoblastoid, diehn-cd28 | 0.79 |
| DPM1 | Dolichyl-phosphate mannosyltransferase polypeptide 1, catalytic subunit | 3 | GSE1133, cheung-lymphoblastoid, diehn-cd28 | 0.79 |
| GLUD1 | Glutamate dehydrogenase 1 | 3 | leung-gastric, diehn-cd28, GDS462 | 0.79 |
| HNRPA3 | Heterogeneous nuclear ribonucleoprotein A3 | 3 | GSE1133, su-human, diehn-cd28 | 0.79 |
| HSPD1 |  | 3 | su-human, diehn-cd28, welsh-prostate | 0.79 |
| LSM3 | LSM3 homolog, U6 small nuclear RNA associated (S. cerevisiae) | 3 | GSE1133, su-human, diehn-cd28 | 0.79 |
| MGC15396 |  | 3 | leung-gastric, chang-fibroblasts, diehn-cd28 | 0.79 |
| NHP2L1 | NHP2 non-histone chromosome protein 2-like 1 (S. cerevisiae) | 3 | GSE1133, diehn-cd28, GDS462 | 0.79 |
| PCMT1 | Protein-L-isoaspartate (D-aspartate) O-methyltransferase | 3 | leung-gastric, su-human, GDS462 | 0.79 |
| SNRPD2 | Small nuclear ribonucleoprotein D2 polypeptide 16.5kDa | 3 | cheok-leukemia, GSE1133, su-human | 0.79 |
| TIAL1 | TIA1 cytotoxic granule-associated RNA binding protein-like 1 | 3 | GSE1133, su-human, diehn-cd28 | 0.79 |
| TXNDC |  | 3 | GSE1133, su-human, diehn-cd28 | 0.79 |
| ZRF1 | Zuotin related factor 1 | 3 | GSE1133, whitfield-cellcycle, diehn-cd28 | 0.79 |
| ATP5C1 | ATP synthase, H+ transporting, mitochondrial F1 complex, gamma polypeptide 1 | 3 | GSE1133, diehn-cd28, welsh-prostate | 0.78 |
| BC066990 |  | 3 | leung-gastric, cheung-lymphoblastoid, diehn-cd28 | 0.78 |
| CPSF6 | Cleavage and polyadenylation specific factor 6, 68kDa | 3 | GSE1133, leung-gastric, diehn-cd28 | 0.78 |
| ILF3 | Interleukin enhancer binding factor 3, 90kDa | 3 | GSE1133, su-human, diehn-cd28 | 0.78 |
| MRPL3 | Mitochondrial ribosomal protein L3 | 3 | GDS564, armstrong-mll, su-human | 0.78 |
| PAICS |  | 3 | su-human, diehn-cd28, welsh-prostate | 0.78 |
| SLC25A6 | Solute carrier family 25 (mitochondrial carrier; adenine nucleotide translocator), member 6 | 3 | GSE1133, luo-prostate, diehn-cd28 | 0.78 |
| SLC35B1 | Solute carrier family 35, member B1 | 3 | luo-prostate, cheung-lymphoblastoid, diehn-cd28 | 0.78 |
| SUMO2 |  | 3 | GSE1133, whitfield-cellcycle, diehn-cd28 | 0.78 |
| VDP | Vesicle docking protein p115 | 3 | sarwal-allograft, GDS558, GSE1133 | 0.78 |
| APPBP1 | Amyloid beta precursor protein binding protein 1 | 3 | GSE1133, su-human, diehn-cd28 | 0.77 |
| C14ORF32 | Chromosome 14 open reading frame 32 | 3 | GSE1133, whitfield-cellcycle, diehn-cd28 | 0.77 |
| C7ORF28A | Chromosome 7 open reading frame 28A | 3 | GSE1133, su-human, diehn-cd28 | 0.77 |
| DDX1 | DEAD (Asp-Glu-Ala-Asp) box polypeptide 1 | 3 | GSE1133, armstrong-mll, diehn-cd28 | 0.77 |
| EIF2S2 |  | 3 | GSE1133, luo-prostate, diehn-cd28 | 0.77 |
| EIF3S10 |  | 3 | GSE1133, leung-gastric, diehn-cd28 | 0.77 |
| FXR1 | Fragile X mental retardation, autosomal homolog 1 | 3 | GDS558, leung-gastric, virtaneva-aml8 | 0.77 |
| HNRPU | Heterogeneous nuclear ribonucleoprotein U (scaffold attachment factor A) | 3 | GSE1133, leung-gastric, diehn-cd28 | 0.77 |
| IMPDH2 | IMP (inosine monophosphate) dehydrogenase 2 | 3 | sarwal-allograft, su-human, diehn-cd28 | 0.77 |
| KIAA0052 |  | 3 | GSE1133, su-human, diehn-cd28 | 0.77 |
| KPNA1 | Karyopherin alpha 1 (importin alpha 5) | 3 | sarwal-allograft, west-breast, cheung-lymphoblastoid | 0.77 |
| MRPS31 | Mitochondrial ribosomal protein S31 | 3 | leung-gastric, GSE1133, GDS462 | 0.77 |
| PRKDC | Protein kinase, DNA-activated, catalytic polypeptide | 3 | shipp-dlbcl, su-human, diehn-cd28 | 0.77 |
| PTMA | Prothymosin, alpha (gene sequence 28) | 3 | GSE1133, su-human, diehn-cd28 | 0.77 |
| RARS | Arginyl-tRNA synthetase | 3 | GSE1133, rickman-glioma, diehn-cd28 | 0.77 |
| SND1 | Staphylococcal nuclease domain containing 1 | 3 | shipp-dlbcl, GSE1133, diehn-cd28 | 0.77 |
| SRP72 | Signal recognition particle 72kDa | 3 | GSE1133, leung-gastric, diehn-cd28 | 0.77 |
| YME1L1 | YME1-like 1 (S. cerevisiae) | 3 | leung-gastric, GSE1133, diehn-cd28 | 0.77 |
| BCLAF1 |  | 3 | GSE1133, whitfield-cellcycle, diehn-cd28 | 0.76 |
| CKS2 | CDC28 protein kinase regulatory subunit 2 | 3 | sarwal-allograft, su-human, diehn-cd28 | 0.76 |
| ME2 | Malic enzyme 2, NAD(+)-dependent, mitochondrial | 3 | GSE1133, armstrong-mll, diehn-cd28 | 0.76 |
| MRPS17 | Mitochondrial ribosomal protein S17 | 3 | GDS558, leung-gastric, GDS462 | 0.76 |
| NOL1 | Nucleolar protein 1, 120kDa | 3 | GSE1133, su-human, diehn-cd28 | 0.76 |
| OAT | Ornithine aminotransferase (gyrate atrophy) | 3 | whitfield-cellcycle, diehn-cd28, hedenfalk-breast | 0.76 |
| PSMD13 | Proteasome (prosome, macropain) 26S subunit, non-ATPase, 13 | 3 | sarwal-allograft, GSE1133, diehn-cd28 | 0.76 |
| RAN |  | 3 | GSE1133, su-human, diehn-cd28 | 0.76 |
| RG9MTD1 | RNA (guanine-9-) methyltransferase domain containing 1 | 3 | GSE1133, whitfield-cellcycle, diehn-cd28 | 0.76 |
| SNRPA | Small nuclear ribonucleoprotein polypeptide A | 3 | GSE1133, su-human, diehn-cd28 | 0.76 |
| UBA2 |  | 3 | leung-gastric, su-human, diehn-cd28 | 0.76 |
| ZNF9 |  | 3 | GSE1133, leung-gastric, whitfield-cellcycle | 0.76 |
| AHCY | S-adenosylhomocysteine hydrolase | 3 | leung-gastric, GDS462, diehn-cd28 | 0.75 |
| AHSA1 | AHA1, activator of heat shock 90kDa protein ATPase homolog 1 (yeast) | 3 | GSE1133, leung-gastric, diehn-cd28 | 0.75 |
| ATIC | 5-aminoimidazole-4-carboxamide ribonucleotide formyltransferase/IMP cyclohydrolase | 3 | ross-nci60, su-human, diehn-cd28 | 0.75 |
| BMS1L | BMS1-like, ribosome assembly protein (yeast) | 3 | GSE1133, su-human, diehn-cd28 | 0.75 |
| BUB3 | BUB3 budding uninhibited by benzimidazoles 3 homolog (yeast) | 3 | leung-gastric, GSE1133, su-human | 0.75 |
| CAPZA1 | Capping protein (actin filament) muscle Z-line, alpha 1 | 3 | GSE1133, su-human, diehn-cd28 | 0.75 |
| COG2 | Component of oligomeric golgi complex 2 | 3 | sarwal-allograft, cheung-lymphoblastoid, huang-breast | 0.75 |
| CPOX | Coproporphyrinogen oxidase | 3 | rinaldi-leukemia, leung-gastric, whitfield-cellcycle | 0.75 |
| DHX9 | DEAH (Asp-Glu-Ala-His) box polypeptide 9 | 3 | GSE1133, leung-gastric, diehn-cd28 | 0.75 |
| EIF2B1 | Eukaryotic translation initiation factor 2B, subunit 1 alpha, 26kDa | 3 | GSE1133, ross-nci60, diehn-cd28 | 0.75 |
| G3BP |  | 3 | GSE1133, leung-gastric, diehn-cd28 | 0.75 |
| LOC134218 |  | 3 | GSE1133, whitfield-cellcycle, diehn-cd28 | 0.75 |
| NDUFS2 | NADH dehydrogenase (ubiquinone) Fe-S protein 2, 49kDa (NADH-coenzyme Q reductase) | 3 | GDS531, sorlie-breasttumor, welsh-prostate | 0.75 |
| NUDT5 | Nudix (nucleoside diphosphate linked moiety X)-type motif 5 | 3 | GSE1133, leung-gastric, cheung-lymphoblastoid | 0.75 |
| NUP50 | Nucleoporin 50kDa | 3 | sarwal-allograft, GSE1133, diehn-cd28 | 0.75 |
| PGK1 | Phosphoglycerate kinase 1 | 3 | leung-gastric, GDS462, diehn-cd28 | 0.75 |
| PNN | Pinin, desmosome associated protein | 3 | GSE1133, su-human, diehn-cd28 | 0.75 |
| PP591 |  | 3 | khatua-astrocytoma, leung-gastric, GSE1133 | 0.75 |
| PSMA7 | Proteasome (prosome, macropain) subunit, alpha type, 7 | 3 | GSE1133, leung-gastric, diehn-cd28 | 0.75 |
| PSMC5 | Proteasome (prosome, macropain) 26S subunit, ATPase, 5 | 3 | GSE1133, diehn-cd28, GDS563 | 0.75 |
| PSME2 |  | 3 | GSE1133, rickman-glioma, diehn-cd28 | 0.75 |
| RNF138 |  | 3 | leung-gastric, GSE1133, diehn-cd28 | 0.75 |
| SLC25A5 | Solute carrier family 25 (mitochondrial carrier; adenine nucleotide translocator), member 5 | 3 | GSE1133, staunton-nci60, diehn-cd28 | 0.75 |
| SNRPD1 | Small nuclear ribonucleoprotein D1 polypeptide 16kDa | 3 | GSE1133, leung-gastric, diehn-cd28 | 0.75 |
| ST13 | Suppression of tumorigenicity 13 (colon carcinoma) (Hsp70 interacting protein) | 3 | GSE1133, leung-gastric, whitfield-cellcycle | 0.75 |
| SUCLA2 | Succinate-CoA ligase, ADP-forming, beta subunit | 3 | leung-gastric, diehn-cd28, GDS462 | 0.75 |
| YWHAQ | Tyrosine 3-monooxygenase/tryptophan 5-monooxygenase activation protein, theta polypeptide | 3 | leung-gastric, GSE1133, diehn-cd28 | 0.75 |
| 39331 |  | 3 | GSE1133, su-human, diehn-cd28 | 0.74 |
| ADIPOR1 | Adiponectin receptor 1 | 3 | leung-gastric, diehn-cd28, GDS462 | 0.74 |
| AF116699 |  | 3 | leung-gastric, whitfield-cellcycle, diehn-cd28 | 0.74 |
| AK2 | Adenylate kinase 2 | 3 | GSE1133, su-human, diehn-cd28 | 0.74 |
| CUL4A | Cullin 4A | 3 | GSE1133, leung-gastric, diehn-cd28 | 0.74 |
| DLD | Dihydrolipoamide dehydrogenase | 3 | shipp-dlbcl, ross-nci60, diehn-cd28 | 0.74 |
| EEF1E1 | Eukaryotic translation elongation factor 1 epsilon 1 | 3 | GSE1133, su-human, diehn-cd28 | 0.74 |
| FUBP1 | Far upstream element (FUSE) binding protein 1 | 3 | leung-gastric, GSE1133, diehn-cd28 | 0.74 |
| HBS1L | HBS1-like (S. cerevisiae) | 3 | leung-gastric, diehn-cd28, GDS462 | 0.74 |
| NCBP2 | Nuclear cap binding protein subunit 2, 20kDa | 3 | GSE1133, leung-gastric, diehn-cd28 | 0.74 |
| PABPC1 | Poly(A) binding protein, cytoplasmic 1 | 3 | leung-gastric, GSE1133, su-human | 0.74 |
| PRPF4B | PRP4 pre-mRNA processing factor 4 homolog B (yeast) | 3 | GSE1133, leung-gastric, diehn-cd28 | 0.74 |
| RNPS1 |  | 3 | GSE1133, leung-gastric, diehn-cd28 | 0.74 |
| TEBP |  | 3 | GSE1133, leung-gastric, diehn-cd28 | 0.74 |
| TXNDC9 | Thioredoxin domain containing 9 | 3 | GSE1133, whitfield-cellcycle, diehn-cd28 | 0.74 |
| CCNH | Cyclin H | 3 | sarwal-allograft, whitfield-cellcycle, su-human | 0.73 |
| EIF3S9 | Eukaryotic translation initiation factor 3, subunit 9 eta, 116kDa | 3 | GSE1133, leung-gastric, diehn-cd28 | 0.73 |
| FLJ20303 |  | 3 | GSE1133, leung-gastric, diehn-cd28 | 0.73 |
| GTF3C3 | General transcription factor IIIC, polypeptide 3, 102kDa | 3 | leung-gastric, GSE1133, diehn-cd28 | 0.73 |
| HNRPR | Heterogeneous nuclear ribonucleoprotein R | 3 | GSE1133, leung-gastric, diehn-cd28 | 0.73 |
| LEREPO4 |  | 3 | GSE1133, leung-gastric, su-human | 0.73 |
| NONO | Non-POU domain containing, octamer-binding | 3 | leung-gastric, GSE1133, diehn-cd28 | 0.73 |
| PAI-RBP1 |  | 3 | leung-gastric, GSE1133, diehn-cd28 | 0.73 |
| PES1 | Pescadillo homolog 1, containing BRCT domain (zebrafish) | 3 | GSE1133, leung-gastric, diehn-cd28 | 0.73 |
| PPP1CB |  | 3 | sarwal-allograft, GSE1133, diehn-cd28 | 0.73 |
| SCYE1 | Small inducible cytokine subfamily E, member 1 (endothelial monocyte-activating) | 3 | GSE1133, leung-gastric, diehn-cd28 | 0.73 |
| SRP9 |  | 3 | leung-gastric, GDS462, diehn-cd28 | 0.73 |
| SSR1 | Signal sequence receptor, alpha (translocon-associated protein alpha) | 3 | GSE1133, leung-gastric, diehn-cd28 | 0.73 |
| SYNCRIP |  | 3 | leung-gastric, GSE1133, diehn-cd28 | 0.73 |
| TNPO1 | Transportin 1 | 3 | leung-gastric, GSE1133, diehn-cd28 | 0.73 |
| CAD | Carbamoyl-phosphate synthetase 2, aspartate transcarbamylase, and dihydroorotase | 3 | leung-gastric, su-human, diehn-cd28 | 0.72 |
| CGI-48 |  | 3 | GSE1133, su-human, diehn-cd28 | 0.72 |
| DYT1 |  | 3 | GSE1133, leung-gastric, diehn-cd28 | 0.72 |
| EIF3S2 | Eukaryotic translation initiation factor 3, subunit 2 beta, 36kDa | 3 | leung-gastric, su-human, diehn-cd28 | 0.72 |
| ERP70 |  | 3 | leung-gastric, luo-prostate, diehn-cd28 | 0.72 |
| HNRPA2B1 | Heterogeneous nuclear ribonucleoprotein A2/B1 | 3 | GSE1133, leung-gastric, su-human | 0.72 |
| SMAD2 |  | 3 | GSE1133, leung-gastric, diehn-cd28 | 0.72 |
| SMARCA5 | SWI/SNF related, matrix associated, actin dependent regulator of chromatin, subfamily a, member 5 | 3 | GSE1133, whitfield-cellcycle, diehn-cd28 | 0.72 |
| SMARCE1 | SWI/SNF related, matrix associated, actin dependent regulator of chromatin, subfamily e, member 1 | 3 | leung-gastric, GSE1133, whitfield-cellcycle | 0.72 |
| SNRPG |  | 3 | GSE1133, ross-nci60, su-human | 0.72 |
| UBE2V2 | Ubiquitin-conjugating enzyme E2 variant 2 | 3 | shipp-dlbcl, leung-gastric, diehn-cd28 | 0.72 |
| XPOT | Exportin, tRNA (nuclear export receptor for tRNAs) | 3 | leung-gastric, su-human, diehn-cd28 | 0.72 |
| C10ORF7 |  | 3 | sarwal-allograft, leung-gastric, diehn-cd28 | 0.71 |
| GHITM | Growth hormone inducible transmembrane protein | 3 | GSE1133, ross-nci60, diehn-cd28 | 0.71 |
| MEP50 |  | 3 | GSE1133, ross-nci60, diehn-cd28 | 0.71 |
| MTHFD2 | Methylenetetrahydrofolate dehydrogenase (NADP+ dependent) 2, methenyltetrahydrofolate cyclohydrolase | 3 | leung-gastric, su-human, diehn-cd28 | 0.71 |
| PPM1G | Protein phosphatase 1G (formerly 2C), magnesium-dependent, gamma isoform | 3 | sarwal-allograft, leung-gastric, diehn-cd28 | 0.71 |
| RAD21 | RAD21 homolog (S. pombe) | 3 | GSE1133, leung-gastric, diehn-cd28 | 0.71 |
| RCN1 | Reticulocalbin 1, EF-hand calcium binding domain | 3 | whitfield-cellcycle, rickman-glioma, diehn-cd28 | 0.71 |
| SFRS9 | Splicing factor, arginine/serine-rich 9 | 3 | leung-gastric, su-human, diehn-cd28 | 0.71 |
| SNRPB | Small nuclear ribonucleoprotein polypeptides B and B1 | 3 | leung-gastric, GSE1133, su-human | 0.71 |
| TBCA | Tubulin folding cofactor A | 3 | GSE1133, leung-gastric, armstrong-mll | 0.71 |
| ACTL6A | Actin-like 6A | 3 | leung-gastric, whitfield-cellcycle, diehn-cd28 | 0.7 |
| AFURS1 |  | 3 | leung-gastric, cheung-lymphoblastoid, diehn-cd28 | 0.7 |
| ANAPC7 | Anaphase promoting complex subunit 7 | 3 | leung-gastric, whitfield-cellcycle, diehn-cd28 | 0.7 |
| APTX | Aprataxin | 3 | leung-gastric, chang-fibroblasts, diehn-cd28 | 0.7 |
| ATP5G3 | ATP synthase, H+ transporting, mitochondrial F0 complex, subunit C3 (subunit 9) | 3 | ross-nci60, diehn-cd28, welsh-prostate | 0.7 |
| BC026067 |  | 3 | leung-gastric, whitfield-cellcycle, diehn-cd28 | 0.7 |
| C1ORF37 | Chromosome 1 open reading frame 37 | 3 | GSE1133, GDS531, su-human | 0.7 |
| NOLC1 | Nucleolar and coiled-body phosphoprotein 1 | 3 | GSE1133, ross-nci60, su-human | 0.7 |
| PAK2 | P21 (CDKN1A)-activated kinase 2 | 3 | leung-gastric, GSE1133, diehn-cd28 | 0.7 |
| PPP2R5C | Protein phosphatase 2, regulatory subunit B (B56), gamma isoform | 3 | leung-gastric, su-human, welsh-prostate | 0.7 |
| PTDSS1 | Phosphatidylserine synthase 1 | 3 | shipp-dlbcl, GSE1133, leung-gastric | 0.7 |
| REA |  | 3 | sarwal-allograft, GDS531, diehn-cd28 | 0.7 |
| RIC-8 |  | 3 | leung-gastric, GSE1133, diehn-cd28 | 0.7 |
| SF3B1 | Splicing factor 3b, subunit 1, 155kDa | 3 | GSE1133, leung-gastric, whitfield-cellcycle | 0.7 |
| SKB1 |  | 3 | leung-gastric, GSE1133, luo-prostate | 0.7 |
| SNRPC | Small nuclear ribonucleoprotein polypeptide C | 3 | GSE1133, leung-gastric, diehn-cd28 | 0.7 |
| TDG |  | 3 | GSE1133, leung-gastric, diehn-cd28 | 0.7 |
| UBL4 |  | 3 | shipp-dlbcl, leung-gastric, GSE1133 | 0.7 |
| VPS26 |  | 3 | GSE1133, leung-gastric, whitfield-cellcycle | 0.7 |
| FLJ20729 |  | 3 | leung-gastric, GSE1133, diehn-cd28 | 0.69 |
| RPL24 |  | 3 | cheok-leukemia, GSE1133, yeoh-leukemia | 0.69 |
| RPP40 | Ribonuclease P 40kDa subunit | 3 | leung-gastric, armstrong-mll, su-human | 0.69 |
| HIP2 | Huntingtin interacting protein 2 | 3 | GSE1133, leung-gastric, diehn-cd28 | 0.68 |
| SF3A3 | Splicing factor 3a, subunit 3, 60kDa | 3 | GSE1133, leung-gastric, diehn-cd28 | 0.68 |
| WTAP | Wilms tumor 1 associated protein | 3 | GSE1133, leung-gastric, whitfield-cellcycle | 0.68 |
| DHX40 | DEAH (Asp-Glu-Ala-His) box polypeptide 40 | 3 | leung-gastric, GSE1133, whitfield-cellcycle | 0.67 |
| GRPEL1 | GrpE-like 1, mitochondrial (E. coli) | 3 | leung-gastric, GSE1133, diehn-cd28 | 0.67 |
| RBM14 |  | 3 | GSE1133, leung-gastric, diehn-cd28 | 0.67 |
| ANP32E | Acidic (leucine-rich) nuclear phosphoprotein 32 family, member E | 3 | GSE1133, leung-gastric, su-human | 0.66 |
| FLJ14668 | Hypothetical protein FLJ14668 | 3 | leung-gastric, ross-nci60, diehn-cd28 | 0.66 |
| IPO9 | Importin 9 | 3 | leung-gastric, ross-nci60, sorlie-breasttumor | 0.66 |
| POLR3C | Polymerase (RNA) III (DNA directed) polypeptide C (62kD) | 3 | armstrong-mll, GDS531, huang-breast | 0.58 |
| VMP |  | 3 | GSE1133, leung-gastric, whitfield-cellcycle | -0.52 |
| SFMBT2 | Scm-like with four mbt domains 2 | 3 | leung-gastric, GDS592, diehn-cd28 | -0.57 |
| ATXN1 | Ataxin 1 | 3 | leung-gastric, west-breast, su-human | -0.58 |
| AB020684 |  | 3 | GDS449, whitfield-cellcycle, GDS479 | -0.79 |
